# Corticotropin-releasing hormone test predicts the outcome of unilateral adrenalectomy in primary bilateral macronodular adrenal hyperplasia

**DOI:** 10.1007/s40618-023-02204-2

**Published:** 2023-10-05

**Authors:** I. Tizianel, M. Detomas, T. Deutschbein, M. Fassnacht, N. Albiger, M. Iacobone, C. Scaroni, F. Ceccato

**Affiliations:** 1 Endocrine Unit, Department of Medicine DIMED, Padua, Italy; 2 Endocrine Unit, University-Hospital of Padova, Padua, Italy; 3 University Hospital, University of Würzburg, Würzburg, Germany, Department of Internal Medicine I, Division of Endocrinology and Diabetes, Würzburg, Germany; 4 Medicover Oldenburg MVZ, Oldenburg, Germany; 5 Radiotherapy Unit, Istituto Oncologico Veneto, Endocrinology, ULSS6 Padua, Euganea Italy; 6 Endocrine Surgery Unit, Department of Surgery, Oncology and Gastroenterology, University of Padova, Padua, Italy

**Keywords:** Cushing’s syndrome, Primary bilateral macronodular adrenal hyperplasia, Unilateral adrenalectomy, hCRH test, Surgical remission

## Abstract

**Purpose:**

Primary bilateral adrenal hyperplasia (PBMAH) is associated with hypercortisolism and a heterogeneous clinical expression in terms of cortisol secretion and related comorbidities. Historically, treatment of choice was bilateral adrenalectomy (B-Adx); however, recent data suggest that unilateral adrenalectomy (U-Adx) may be an effective alternative. For the latter, factors predicting the postsurgical outcome (e.g., biochemical control) have not been identified yet.

**Methods:**

PBMAH patients undergoing U-Adx for overt Cushing’s syndrome (CS) in two tertiary care centers were retrospectively analysed. Remission was defined as a normalization of urinary free cortisol (UFC) without the need for medical treatment. The potential of hCRH test as a predictor of U-Adx outcome was evaluated in a subgroup.

**Results:**

23 patients were evaluated (69% females, mean age 55 years). Remission rate after U-Adx was 74% at last follow up (median 115 months from UAdx). Before U-Adx, a positive ACTH response to hCRH (Δ%ACTH increase > 50% from baseline) was associated with higher remission rates.

**Conclusions:**

Three of four patients with PBMAH are surgically cured with U-Adx. Pre-operative hCRH testing can be useful to predict long-term remission rates.

**Supplementary Information:**

The online version contains supplementary material available at 10.1007/s40618-023-02204-2.

## Introduction

Primary bilateral macronodular adrenal hyperplasia (PBMAH) is a rare condition characterized by bilateral adrenal enlargement with benign macro-nodules larger than 1 cm [1]. These macro-nodules are oversecreting cortisol in different degrees, therefore, resulting in a heterogeneous biochemical disease expression ranging from autonomous cortisol secretion (ACS) to overt Cushing’s syndrome (CS). Nowadays, a large number of patients with PBMAH are often incidentally detected during abdominal imaging performed for other reasons [2].

The amount of cortisol secretion can suppress the hypothalamus: a low ACTH is the hallmark of overt adrenal secretion. In some cases, ACTH-dependent adrenal macro-nodules can also occur in patients with Cushing’s disease [3], low-normal ACTH levels are not able to completely differentiate ACTH-dependent from ACTH-independent hyperocortisolism [4]. Therefore, dynamic testing with human corticotropin-releasing hormone (hCRH) has been suggested to confirm ACTH-independent adrenal disease [5, 6].

Medical or surgical treatment has to be considered in cases of overt CS or ACS associated with cortisol-related comorbidities [2].

Historically, bilateral adrenalectomy has been proposed as the treatment of choice in PBMAH patients with overt CS [2, 7]. However, despite the efficiency in controlling hypercortisolism, it implies lifelong gluco- and mineralocorticoid replacement, with the risk of developing relevant morbidity and mortality (e.g., due to adrenal crisis) [7].

More recently, unilateral adrenalectomy (U-Adx) has been proposed as an alternative treatment, especially in PBMAH patients with less severe hypercortisolemia and volumetric adrenal asymmetry in computed tomography (CT) scans [8, 9]. The decision for the individual surgical approach takes adrenal size and certain imaging features into account. Usually, the larger gland is removed, particularly if it comes along with a higher uptake of cholesterol or 18-FDG in adrenal scintigraphy or position tomography (PET) [10].

Medical treatment with steroidogenesis inhibitors (i.e., metyrapone or ketoconazole) is suggested in case of contraindication to surgery, mild ACS without relevant cardiovascular impairment, or as a “bridging therapy” until surgery.

Two genetic cause of PBMAH has been demonstrated: constitutive inactivating variants in ARMC5 and KDM1A [11, 12]. Of note, *ARMC5* mutations are present in about 80% of familiar cases [11, 13], and in about 20–25% of all PBMAH patients [14, 15]. The presence of an *ARMC5* mutation seems to be associated with more adrenal nodules, an increased adrenal size, a more severe disease course, and worse cortisol-related comorbidities. Accordingly, ARMC5 mutated patients require surgical or medical treatment more frequently [2, 16].

This study evaluates the role of U-Adx in PBMAH patients and the ACTH responses to hCRH, thereby aiming to identify possible predictive factors for the surgical outcome of U-Adx for the first time.

## Patients and methods

Twenty-three PBMAH patients who underwent U-Adx for overt CS in two tertiary care centers (Padua, Italy; Würzburg, Germany) were retrospectively identified, taking the time from 2001 to 2019 into account.

Of note, 10 out of 23 patients of our cohort were previously described in the study of Albiger et al., whose follow-up lasted until 2015 [17].

Informed consent was collected from all patients, and the study protocol was approved by the local Ethic Committee of the University Hospital of Padova (protocol number 0042129 of 2023).

Overt CS was defined as urinary free cortisol (UFC) level > 2 times the upper limit of normality (ULN) and an unsuppressed serum cortisol after an overnight 1-mg dexamethasone suppression test (DST) (i.e., serum cortisol > 50 nmol/L). The diagnosis of bilateral macronodular hyperplasia was based on the morphological appearance during computed tomographic or magnetic resonance imaging and subsequent histological confirmation.

Clinical characteristics at baseline and last follow-up are shown in Table 1. 16/23 patients (69%) were female, mean age at initial diagnosis was 55 years (range), and median follow-up duration was 115 months (18–326 months). Data about cortisol-related comorbidities (i.e., arterial hypertension, diabetes mellitus, obesity, osteoporosis, cardiovascular complications) and first line biochemical testing for CS (i.e., UFC, serum cortisol after overnight 1-mg DST, late night salivary cortisol (LNSC)), and ACTH levels were recorded preoperatively and again periodically after surgery (including the last follow-up visit).

**Table 1:** Descriptive analysis of BMAH^remission^ and BMAH^active^ groups

	BMAH^remission^	BMAH^active^	*p* value
Clinical characteristics			
Gender (F/M)	11/6	5/1	n.s (0.621)
Age at initial diagnosis (median)	57	50	n.s (0.417)
ARMC5 mutational status			
Wild type	10	2	n.s (0.611)
Mutated	6	3	
Biochemistry at initial diagnosis			
DST			
Serum cortisol 50–138 nmol/L	1	0	n.s (1.000)
Serum cortisol > 138 nmol/L	9	4	
UFC			
> 2 ULN	3	2	n.s (1.000)
> 3 ULN	7	3	
Dynamic testing with hCRH			
Positive response (Δ%ACTH > 50%)	8	0	0.001
Negative response (Δ%ACTH < 50%)	1	6	
Side of U-Adx			
Left	8	3	n.s (1.000)
Right	7	3	

Abbreviations: *BMAH* bilateral macronodular adrenal hyperplasia, *DST* dexamethasone suppression test, U-Adx unilateral adrenalectomy, *UFC* urinary free cortisol

ARMC5 mutational status (2 missing cases), DST (9 missing cases), UFC (8 missing cases), hCRH (8 missing cases), side of U-Adx (2 not known)

A subgroup of patients (n = 15) underwent a hCRH test before U-Adx. This test was performed in the morning (i.e., 8.00–9.00 a.m.), after overnight fasting. After baseline sampling (at − 15 and 0 min), further blood samples for ACTH and cortisol measurement were collected − 15, 30, 45, 60, 90, and 120 min after an intravenous bolus injection of 100 μg hCRH. A positive ACTH response to hCRH infusion was considered as a Δ%ACTH increase > 50% from baseline. Other dynamic tests (especially desmopressin test), imaging (as pituitary magnetic resonance) and long-term follow-up were used to confirm adrenal source of CS in case of positive CRH response.

All patients underwent U-Adx with the aim to control overt hypercortisolism and to improve cortisol-related comorbidities. The decision on which adrenal should be removed was mainly based on adrenal size (with the largest adrenal being removed). In a few cases, these data have been integrated with NP-59 scintigraphy uptake.

After U-Adx, PBMAH patients were periodically re-evaluated according to common clinical practice.

Remission was considered if the following features were fulfilled: UFC < ULN, no worsening of cortisol-related comorbidities, no need for medical treatment with steroidogenesis inhibitors. In this case, patients were stratified into the BMAH^remission^ group. Patients with persistent hypercortisolism were defined as presence of UFC > ULN, the need for further therapy (either medical therapy or B-Adx). These subjects were stratified into the BMAH^active^group. Both patient groups (i.e., BMAH^remission^ vs BMAH^active^) were compared regarding gender, age, ARMC5 mutational status, and the outcome to both preoperative hCRH testing and adrenal surgery.

A descriptive analysis was performed, using frequencies, means, and dispersion measures. Data between the two groups were compared using the X2 test for qualitative variables (or Fisher’s exact test when the cell count was < 5) and the Mann–Whitney *U* test for quantitative ones. A *p* value < 0.05 was considered statistically significant (two-sided tests). The statistical analysis was performed using SPSS software version 24.

## Results

At the last follow up after U-Adx, BMAH^remission^ group was made of 17/23 patients (74%), while BMAH^active^ group consisted of 6/23 patients (26%).

**Fig. 1:**
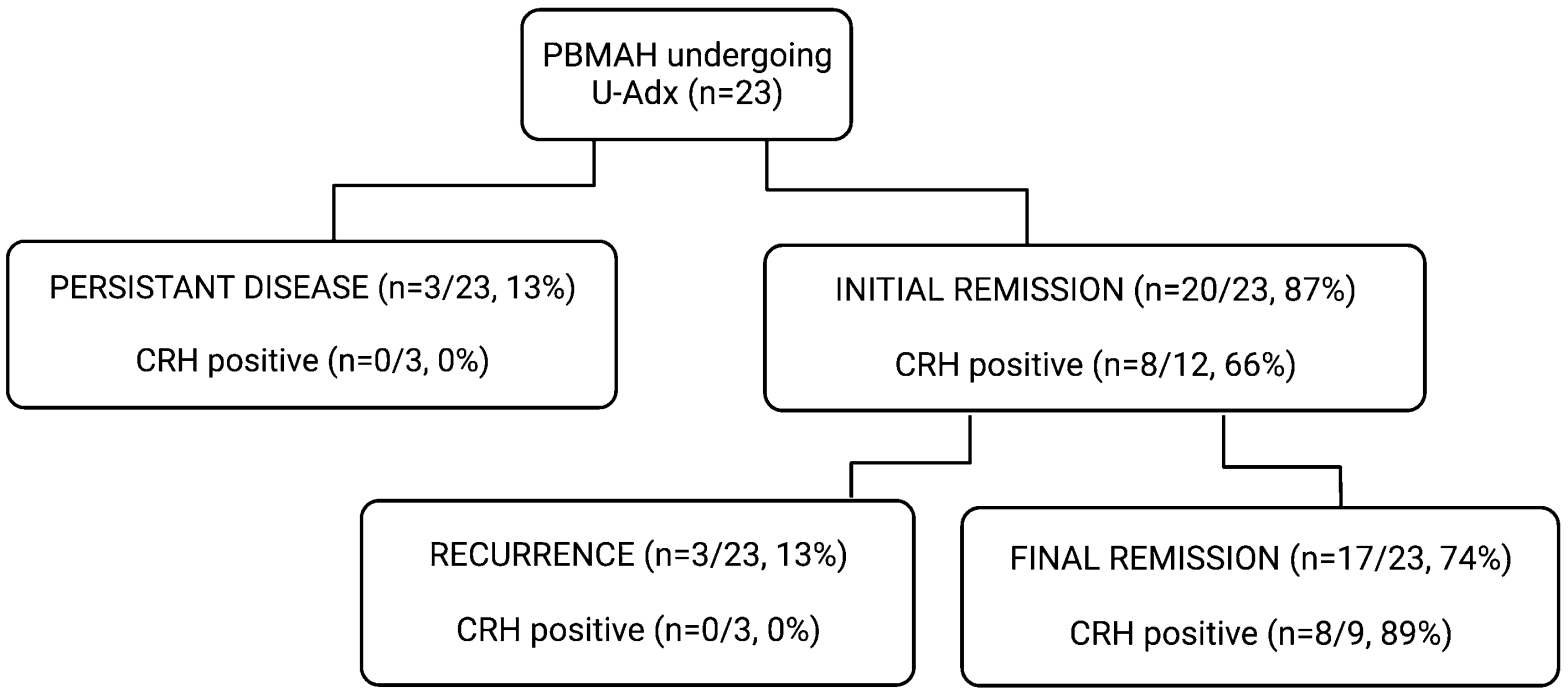
Clinical outcome and ACTH response to the preoperative hCRH test. Remission is defined by normalization of urinary free cortisol without needing medical therapy for hypercortisolism

Among the latter, three patients experienced recurrent disease after initial remission (respectively, 11, 24, and 26 months after U-Adx). The other three patients had persistent hypercortisolism after surgery. The study cohort is depicted in Fig. 1.

ARMC5 status was available in 21/23 patients (91%): 9/21 (43%) were carriers of an *ARMC5* mutation, while 12/21 (57%) were wild type.

Both study groups (BMAH^remission^, BMAH^active^) were well-comparable regarding gender, age at initial diagnosis, degree of hypercortisolism at initial diagnosis (considering UFC and cortisol after 1 mg-DST), ARMC5 mutational status, and side of U-Adx (as shown in Table 1). At initial diagnosis, 20/23 patients (87%) had arterial hypertension and 8/23 (35%) had diabetes mellitus (similar prevalence to literature, as reported in Supplementary Table 1).

Fifteen patients (15/23 = 65%) underwent hCRH test before U-Adx, depicted in Supplementary Table 2. In this subgroup, the relationship between ACTH responses to hCRH injection and surgical outcome were compared (BMAH^remission^, *n* = 9; BMAH^active^, *n* = 6). No ACTH response to hCRH was observed in 7/15 patients (47%).

**Fig. 2:**
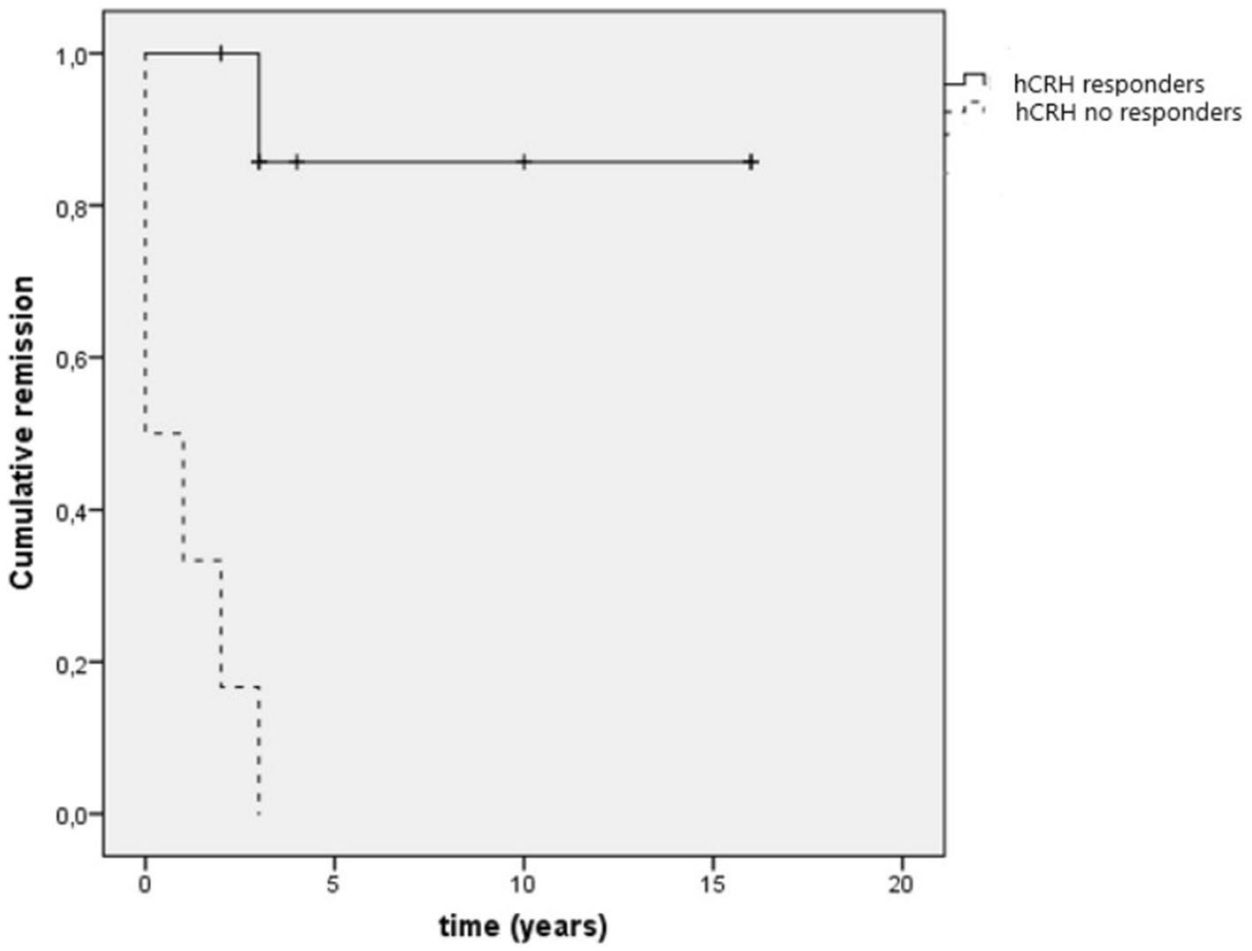
Kaplan–Meier’s outcome after hCRH test

None of the patients in the BMAH^active^ group had a positive response to hCRH. Among them, 3 patients did not experience remission after U-Adx and the other 3 experienced recurrent disease after initial remission (accordingly, persistent hypercortisolism at last follow-up was found in 6 of 7 PBMAH patients who did not respond to the preoperative hCRH test). The other 8 patients showed a positive ACTH response to hCRH, all of them experiencing remission after U-Adx. Long-term remission was more pronounced in the group with positive ACTH response to hCRH than in non-responders (as summarized in Fig. 2). Of note, both the positive and the negative responders to hCRH were well-comparable regarding gender, age, degree of initial hypercortisolism, and ARMC5 mutational status (as illustrated in Table 2).

**Table 2:** Descriptive analysis of hCRH positive and hCRH negative groups

	hCRH positive response (Δ% ACTH > 50%)	hCRH negative response (Δ% ACTH < 50%)	*p* value
Clinical characteristics			
Gender (F/M)	5/3	6/1	n.s (0.569)
Age at initial diagnosis (median)	59	51	n.s (0.340)
ARMC5 mutational status			
Wild type	5	3	n.s (0.592)
Mutated	2	3	
Endocrine evaluation at initial diagnosis			
DST			
Serum cortisol 5–138 nmol/L	0	0	n.s (1.000)
Serum cortisol > 138 nmol/L	6	5	
UFC			
> 2 ULN	3	2	n.s (0.567)
> 3 ULN	2	4	
Follow up			
Months (median)	39	78	n.s (0.378)

Abbreviations: *DST* dexamethasone suppression test, *U-Adx* unilateral adrenalectomy, *UFC* urinary free cortisol; ARMC5 mutational status (2 missing cases), DST (4 missing cases) UFC (4 missing cases)

We also compared ACTH levels before and after U-Adx between the two study groups (BMAH^remission^ and BMAH^active^).

**Fig. 3:**
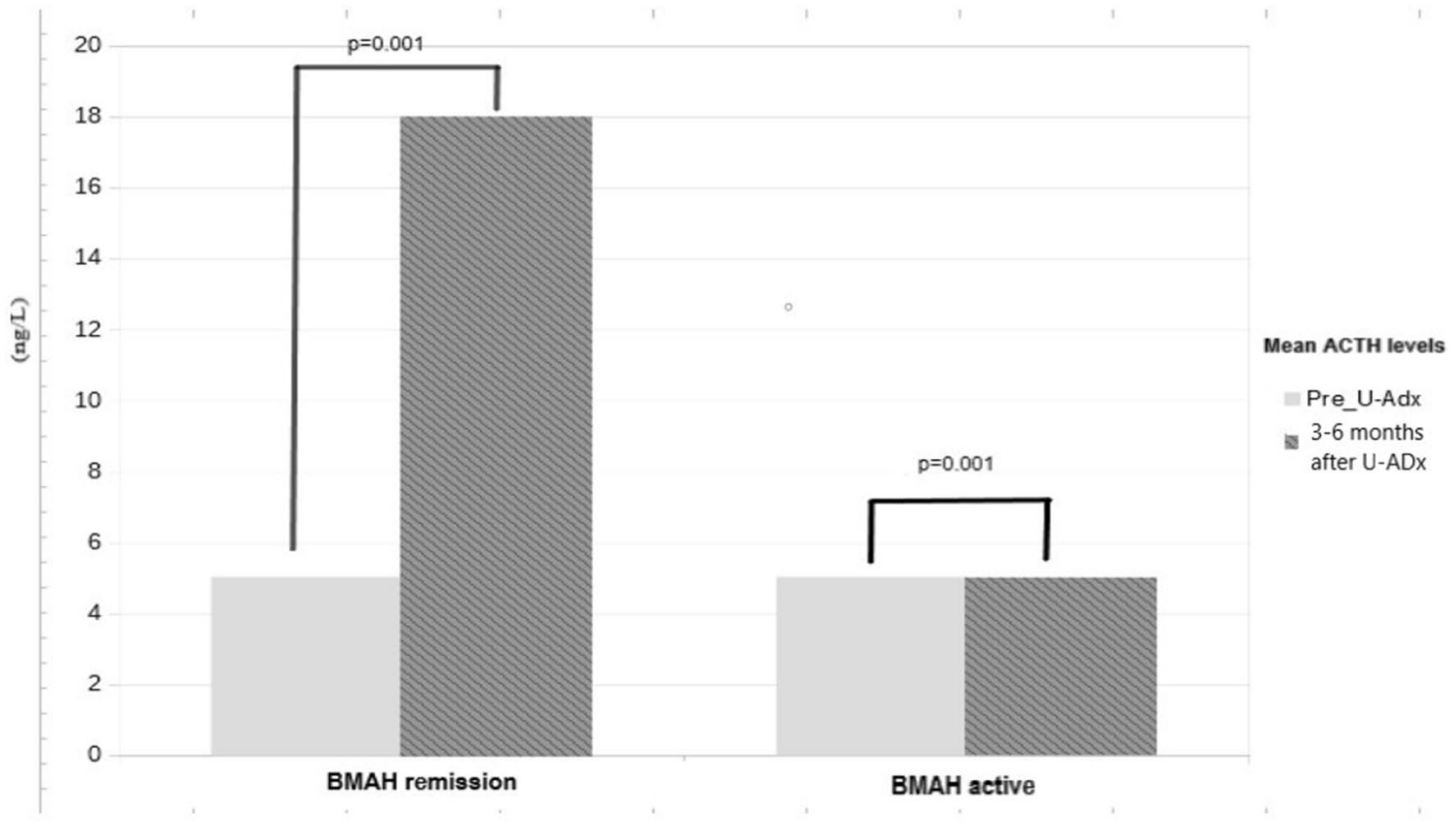
Baseline ACTH levels before and after U-Adx

A significant difference between baseline and post-operative ACTH levels was only observed in patients experiencing surgical remission (*p* = 0.001, resumed in Fig. 3). Accordingly, a post-surgical rise of ACTH levels is a marker of HPA axis recovery, whereas persistent disease results in low or undetectable ACTH levels.

## Discussion

PBMAH is biochemically and clinically a very heterogeneous disease. Therefore, a personalized treatment approach is required [1, 2]. According to guidelines and consensus panels [18] PBMAH patients with overt CS have to be surgically treated, whereas patients with clinically unapparent hypercortisolism could be monitored conservatively until worsening of comorbidities or development of hypercortisolism.

Despite good remission rates even in case of overt CS (reassumed in supplementary Table 3), a recent retrospective study showed an increased mortality in patients treated by U-Adx compared to those who underwent B-Adx. This observation is probably most likely related to a less pronounced control of hypercortisolism after U-Adx [19]. Consequently, some authors still suggest B-Adx in cases of severe CS and symmetry of the adrenal glands.

In PBMAH, steroidogenesis is regulated by the aberrant expression of G-protein coupled receptors (GPCR), which activate cAMP/PKA pathway through their different ligands [20, 21]. This has opened the alternative to the use of target medical treatments with specific antagonists but with limited efficacy [2, 17].

Until now, no factors have been identified to predict U-Adx outcome to control hypercortisolism and to reduce the risk of persistent/recurrent disease. Several studies demonstrated that in case of asymmetric adrenal size, a favourable surgery outcome was obtained if the largest adrenal was removed [10, 17].

In the largest series published so far, higher baseline UFC levels and contralateral residual adrenal volume > 34 ml were considered associated with a higher risk for recurrence. Contralateral adrenalectomy was required in 55% of patients, particularly among those with mean UFC levels > 5.8 times ULN (illustrating more pronounced CS) [22].

In a recent prospective study, 10/17 BMAH patients with overt CS were treated with resection of the largest adrenal and a partial sparing contralateral adrenalectomy. After surgery, 95% of these patients achieved remission of hypercortisolism [23].

ARMC5 mutations are due to a biallelic inactivation that is compatible to a tumour suppressor gene model (germline and subsequent somatic mutation). Therefore, respective genetic alterations are present in both adrenal glands [18]. Accordingly, it appears reasonable to refer affected patients to B-Adx as first line treatment. In our patient cohort, however, there were no differences between ARMC5-mutated and wild-type patients in terms of CS remission after U-Adx. Therefore, further studies on these aspects would be of great interest.

To identify possible predictive factors for the surgical outcome of U-Adx, we put special emphasis on the preoperative hCRH test, this test is commonly used in clinical practice, allowing to assess the entirety of the hypothalamic–pituitary–adrenal (HPA) axis [24, 25]. In the past hCRH was routinely performed in adrenal forms of CS, as indicated in the diagnostic work-up of BMAH patients [26, 27]. According to this, we continued to perform hCRH test in our clinical practice, only in bilateral adrenal forms, to rule out an ACTH-dependency, since some cases of CD can present with bilateral adrenal adenomas or with low ACTH levels. The hCRH test is carried out not only as part of the differential diagnosis of ACTH-dependent CS (Cushing disease vs Ectopic ACTH secretion [28, 29]), but also can be used to distinguish between in ACTH-independent CS [30]. As we already demonstrated in a recent study [30], the hCRH test was accurate in distinguishing overt CS from mild ACS in patients with adrenal lesions, since the hCRH-stimulated ACTH and cortisol peak and percentage increases were blunted in case of overt hypercortisolism, but not in mild ACS.

As reported by Pecori Giraldi et al. the evaluation of ACTH levels by commercially available immunoassay is inaccurate in the low-normal range (i.e., below 20 pg/mL). Thus, the hCRH test can confirm the adrenal source of CS (in case of absent ACTH response) or unmask the ACTH-responsiveness of ACTH-dependent CS (e.g., in ACTH-secreting pituitary lesions with concomitant adrenal nodules which become autonomous) [4]. Of note, a relevant shortage of hCRH has recently been reported [31].

In this study, we aimed to study the role of hCRH test in PBMAH with overt CS. We observed that a positive ACTH response to hCRH injection is associated with higher remission rates compared to cases without ACTH response. This may be related to the fact HPA axis integrity is not relevantly affected by moderate hypercortisolism. On the contrary, an absence of ACTH response to hCRH highlights the autonomous nature of adrenal hypercortisolism that may relapse after surgery more easily (or even does not allow for immediate post-operative remission).

To date, it is not known whether there is a greater overall risk related to CS or adrenal insufficiency derived from B-Adx. Therefore, our current study (and hopefully also future studies in this field) may pave the way for possible predictive factors of surgery outcome.

The main limitations of this study are its retrospective nature, the potential risk of a selection bias, and the small number of cases (although it has to be recognized that PBMAH is a very rare disease). Unfortunately, there were few patients with available hCRH test results, since some patients were evaluated even before the 2000s. Moreover, in some cases the test's results (i.e., positive ACTH response) were recorded in our clinical record but not available in terms of detailed ACTH concentrations (and, therefore, were not used).

In conclusion, the remission rate of 74% after U-Adx was similar to published data. As the hCRH test allows a first prediction of the U-Adx outcome, we suggest this test as part of the preoperative diagnostic workup of PBMAH patients undergoing U-Adx.

### Supplementary Information

Below is the link to the electronic supplementary material.

Supplementary file1 (PDF 124 KB)

## Data Availability

The database is available upon reasonable request.
