# Supplementary material for: Corticotropin-releasing hormone test predicts the outcome of unilateral adrenalectomy in primary bilateral macronodular adrenal hyperplasia
Source: J Endocrinol Invest. 2023 Oct 5;47(3):749–56. doi: 10.1007/s40618-023-02204-2 (PMC10904413; doi:10.1007/s40618-023-02204-2)
Supplement: Supplementary file 1 — Supplementary file1 (PDF 124 KB) [file 40618_2023_2204_MOESM1_ESM.pdf]

Corticotropin-releasing hormone test predicts the outcome of unilateral adrenalectomy in Primary Bilateral Macronodular Adrenal Hyperplasia

Authors: Irene Tizianel[1,2], Mario Detomas[3], Timo Deutschbein[3,4], Martin Fassnacht[3], Nora Albiger[5], Maurizio Iacobone[6], Scaroni Carla[1], Filippo Ceccato[1,2]

[1]Endocrine Unit-Department of Medicine DIMED, Padova, Italy
[2]Endocrine Unit, University-Hospital of Padova, Italy
[3]University Hospital, University of Würzburg, Würzburg, Germany, Department of Internal Medicine I, Division of Endocrinology and Diabetes, Würzburg, Germany,
[4]Medicover Oldenburg MVZ, Oldenburg, Germany
[5]Radiotheray Unit, Istituto Oncologico Veneto; Endocrinology, ULSS6 Euganea, Padova, Italy
[6]Endocrine Surgery Unit, Department of Surgery, Oncology and Gastroenterology, University of Padova

Supplementary material

Supplementary Table 1. Prevalence of arterial hypertension and diabetes mellitus in PBMAH patients (study is indicated in the first column, only series with at least 10 patients included).

	**Patients (n)**	**Prevalence**	
		**Arterial hypertension**	**Diabetes mellitus**
Debillon et al, 2015	15 (overt CS: 100%)	67%	30%
Espiard et al, 2015	98 (overt CS: 43%)	69%	34%
Albiger et al., 2017	72 (overt CS: 47%)	67%	24%
Bouys et al., 2022	352 (overt CS: 27%)	73%	38%
Wang et al, 2022	124 (overt CS: 55%)	91%	47%

Supplementary Table 2. Description of patients who underwent hCRH test. PAD: patients from Padova, WUR: patients from Würzburg

	**Baseline ACTH during hCRH (ng/L)**	**Peak ACTH during hCRH (ng/L)**	**Outcome**	**Follow up (months)**	**Size of the removed adrenal lesion**	**Size of contralateral adrenal lesion**	**Scintigraphic uptake**	**Histology**
**PAD 1**	<5	<5	Persistence	160	Left, 5 cm	4.5 cm		Diffuse macronodular cortical hyperplasia
**PAD 2**	5	15	Remission	180	Left, 7 cm	4.5 cm		Adrenal cortical hyperplasia with multiple macronodules
**PAD 3**	<5	<5	Persistence	88	Right, 5.5 cm	2.1 cm e 1.2 cm	Bilateral, with a right predominance	Cortical adrenal nodules (fasciculata cells) with high lipid content
**PAD 4**	6	19	Remission	326	Left, 5.5 cm	5 cm		Diffuse macronodular cortical hyperplasia
**PAD 5**	<5	<5	Persistence	78	Right, 4.5 cm	3.5 cm		Adrenal cortical adenoma with prevalence of fasciculata and reticularis cells
**PAD 6**	4	8	Remission	37	Right, 3.1 cm	1.7 cm	Unilateral right	Adrenal cortical adenoma with prevalence of fasciculata and reticular cells
**PAD 7**	<5	<5	Persistence	26	Left, 3.8 cm	1.6 cm	Bilateral, with a left predominance	Diffuse macronodular cortical hyperplasia
**PAD 8**	5	12	Remission	21	Left, 4.5 cm	3.8 cm	Bilateral, with a strong left predominance	Diffuse macronodular cortical hyperplasia
**PAD 9**	<5	2	Persistence	64	Left, 5.8 cm			Adrenal cortical adenoma with prevalence of fasciculata and reticularis cells

**WUR 1**	<5	15.8	Remission	41	Left, 4.5 cm	2.3 cm	-	Adrenocortical adenoma
**WUR 2**	<5	<5	Persistence	172	Left, 2.7 cm	2.0 cm	-	Diffuse macronodular cortical hyperplasia
**WUR 3**	8.1	23.7	Remission	115	Right, 2.8 cm	2.3 cm	-	Diffuse macronodular cortical hyperplasia
**WUR 4**	7.2	47.8	Remission	18	Left, 4.2 cm	2.5 cm	Bilateral, with a left predominance	Diffuse macronodular cortical hyperplasia
**WUR 5**	<5	<5	Remission	36	Left, 3.7 cm	2.6 cm	-	Diffuse macronodular cortical hyperplasia
**WUR 6**	<5	11.6	Remission	31	Right, 6.4 cm	2.2 cm		Adrenocortical adenoma

SupplementaryTable 3. Outcome of monolateral adrenalectomy in patients with PBMAH (study is indicated in the first column, only series with at least 10 patients included)

	**N° of patients**	**Severity of CS**	**Choice of adrenal to be removed**	**Initial remission (%)**	**Recurrence (%)**	**Final bilateral Adx**	**Median follow up (months)**
Xu et al, 2013	14	overt	largest	13 (93%)	-	1 (7%)	69
Albiger et al, 2015	12	overt 3, mil 5 mild 3 cyclic 1	largest and radioiodine uptake of scintigraphy	11 (92%)	8 (73%)	4 (33%)	92
*Current series **	23	overt	largest and radioiodine uptake of scintigraphy	17 (74%)	3 (13%)	8 (35%)	115
Debillon et al, 2015	15	overt 11 mild 11	largest	15 (100%)	2 (13%)	1 (7%)	60
Li and Yang., 2015	15	overt 13 mild 2	-	15 (100%)	3 (20%)	3 (20%)	-
Osswald et al, 2019	25	overt and mild	largest, cortisol gradient during AVS or scintigraphic uptake	21 (84%)	3 (14%)	3 (14%)	50
Zhang et al, 2020	39	overt 28 mild 11	largest	15/28 (68%) 6/11 (86%)	-	7 overt (32%) 1 mild (14%)	20
Wang, 2022	124	overt 65 mild 56	largest	43/65 (71%) in overt	47/65 (72%) in overt 17/51 (33%) in mild forms	55% (64/116)	28

*The series of Albiger et al., 2015, is partially included in the current study
